# Motor Recovery in Glioma Patients After Craniotomy: A Case Study of Continuous Rehabilitation Assessed With Diffusion Tensor Imaging

**DOI:** 10.7759/cureus.82747

**Published:** 2025-04-21

**Authors:** Yoichiro Horikawa, Takuma Yuri, Chinatsu Umaba, Rie Yamawaki, Manabu Nankaku, Ryosuke Ikeguchi, Yoshiki Arakawa

**Affiliations:** 1 Rehabilitation Unit, Kyoto University Hospital, Kyoto, JPN; 2 Department of Occupational Therapy, Faculty of Health Science, Kyoto Tachibana University, Kyoto, JPN; 3 Department of Rehabilitation Medicine, Kyoto University Hospital, Kyoto, JPN; 4 Department of Neurosurgery, Kyoto University, Kyoto, JPN

**Keywords:** diffusion tensor imaging (dti), edema, fractional anisotropy (fa), motor function deficits, mri, postoperative rehabilitation

## Abstract

Motor function deterioration is a common postoperative complication in glioma patients, affecting daily activities. Although continuous rehabilitation is essential for motor recovery, the underlying cause of postoperative decline remains unclear. This case study investigates the temporal relationship between motor function, corticospinal tract (CST) fractional anisotropy (FA), and edema in a glioma patient following awake craniotomy.

A 43-year-old Japanese man with recurrent left parietal glioma and right spastic hemiparesis underwent seven tumor resections over 12 years, culminating in an awake craniotomy. He experienced postoperative motor decline and underwent continuous rehabilitation. FA values of the CST and motor function were assessed preoperatively and on postoperative days 12, 29, and 134. Magnetic resonance imaging (MRI), T2 fluid-attenuated inversion recovery (FLAIR) was used to monitor edema progression.

On postoperative day 12, a significant increase in edema was observed in the frontoparietal region, coinciding with a decline in FA and motor function. However, by postoperative days 29 and 134, edema had decreased, and both FA and motor function improved. The findings suggest that vasogenic edema contributed to the transient motor decline, as evidenced by the reversible FA changes, suggesting that CST integrity assessment via FA and edema monitoring may guide postoperative rehabilitation strategies.

## Introduction

Motor function in glioma patients often deteriorates post-craniotomy, significantly impacting daily activities and quality of life. Continuous postoperative rehabilitation plays a crucial role in restoring motor function [1-3]. However, to optimize rehabilitation strategies, clinicians must understand the underlying causes of motor function decline.

Previous studies indicate that motor function deterioration is associated with corticospinal tract (CST) alterations due to vasogenic or peritumoral edema near the CST, as observed through magnetic resonance imaging (MRI) and diffusion tensor imaging (DTI) [4-7]. Vasogenic or peritumoral edema typically appears as a high-intensity area on MRI T2 fluid-attenuated inversion recovery (FLAIR) [5,6], while CST integrity can be objectively assessed using fractional anisotropy (FA) values in DTI [7]. In stroke patients, FA values and edema near the CST correlate with motor recovery [8,9]. In addition, continuous rehabilitation is associated with improvements in FA and motor function [10,11]. However, no studies have examined temporal changes in edema, FA values, and motor function in glioma patients following craniotomy.

This case study reports a patient experiencing postoperative motor function decline who underwent continuous rehabilitation while being monitored for CST and edema changes. We hypothesize that serial evaluation of CST and edema can provide insights into rehabilitation strategies and their effects.

## Case presentation

A 43-year-old Japanese man with right spastic hemiparesis due to recurrent left parietal lobe glioma underwent seven tumor resections over 12 years. He was hospitalized for nine days due to progressive hemiparesis and was re-admitted 15 days post-discharge for worsening symptoms. Six days later, he underwent awake craniotomy.

Preoperatively, the patient received intravenous betamethasone (4 mg) and glycerol (200 mL) twice daily for one to six days before surgery, and betamethasone (2 mg) once daily on postoperative days 1-2. He was on levetiracetam (2000 mg), perampanel (8 mg), and clobazam (20 mg) throughout hospitalization and post-discharge. The patient was discharged on postoperative day 30, with regular brain imaging follow-up.

FA of the CST and motor function were evaluated 25 days before craniotomy and on postoperative days 12, 29, and 134. FA was measured using automated region-of-interest (ROI) analysis (Brainlab, Munich, Germany), with the relative FA (rFA) calculated as the FA ratio between the affected and unaffected sides. Motor function was assessed using grip strength (GS) and the Box and Block Test (BBT) [12], with scores converted to Z-scores based on normative data [13].

During hospitalization and after discharge, the patient engaged in continuous rehabilitation based on modified constraint-induced movement therapy (mCIMT) [14], aimed at enhancing motor function through frequent use of the affected upper limb. The rehabilitation goal, with the patient's consent, was to enable him to hang out the laundry using his paralyzed hand in daily life. Rehabilitation sessions were conducted for 20 minutes per session, five times a week, from postoperative day 1 to day 30. In addition to mCIMT, stretching and neuromuscular training were also incorporated to improve upper limb function with supervision from an occupational therapist. These interventions included task-oriented training to improve proximal upper limb muscle strength, hand GS, and hand dexterity. As upper limb function recovered, the patient was encouraged to use their paralyzed hands in daily living in the hospital. Post-discharge, the patient continued self-exercises at home. The home rehabilitation program was assessed during the day 134 follow-up by interviewing the patient and his family, confirming that the frequency and content were maintained at a level comparable to those performed in the hospital due to his high motivation.

**Figure 1:**
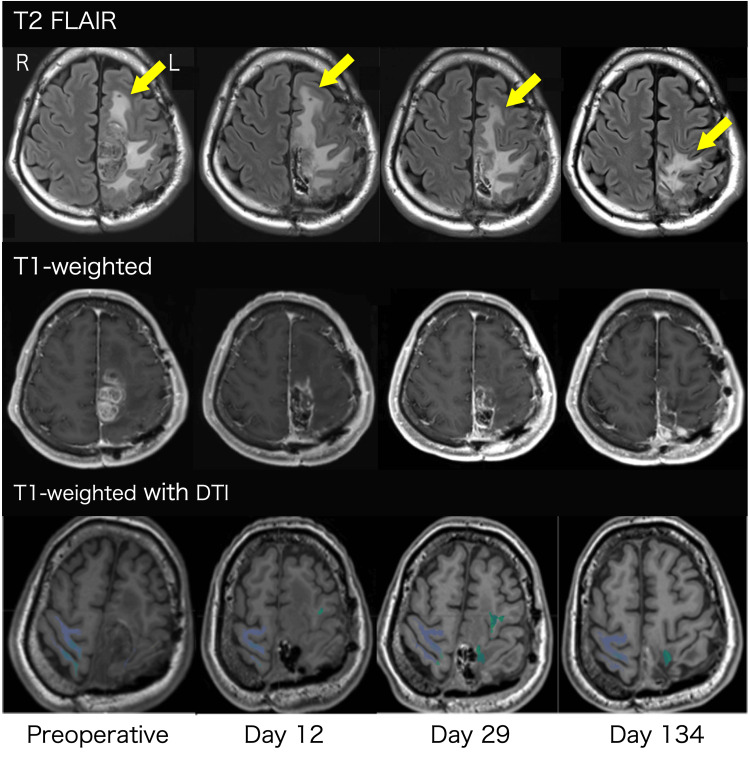
MRI T2 FLAIR high-intensity areas (yellow arrow), tumor regions in T1-weighted images, and fractional anisotropy (FA) of the corticospinal tract (CST) Preoperative evaluation was conducted 25 days before surgery. The colored area on T1-weighted images with diffusion tensor imaging (DTI) indicates the CST. FLAIR: fluid-attenuated inversion recovery

**Figure 2:**
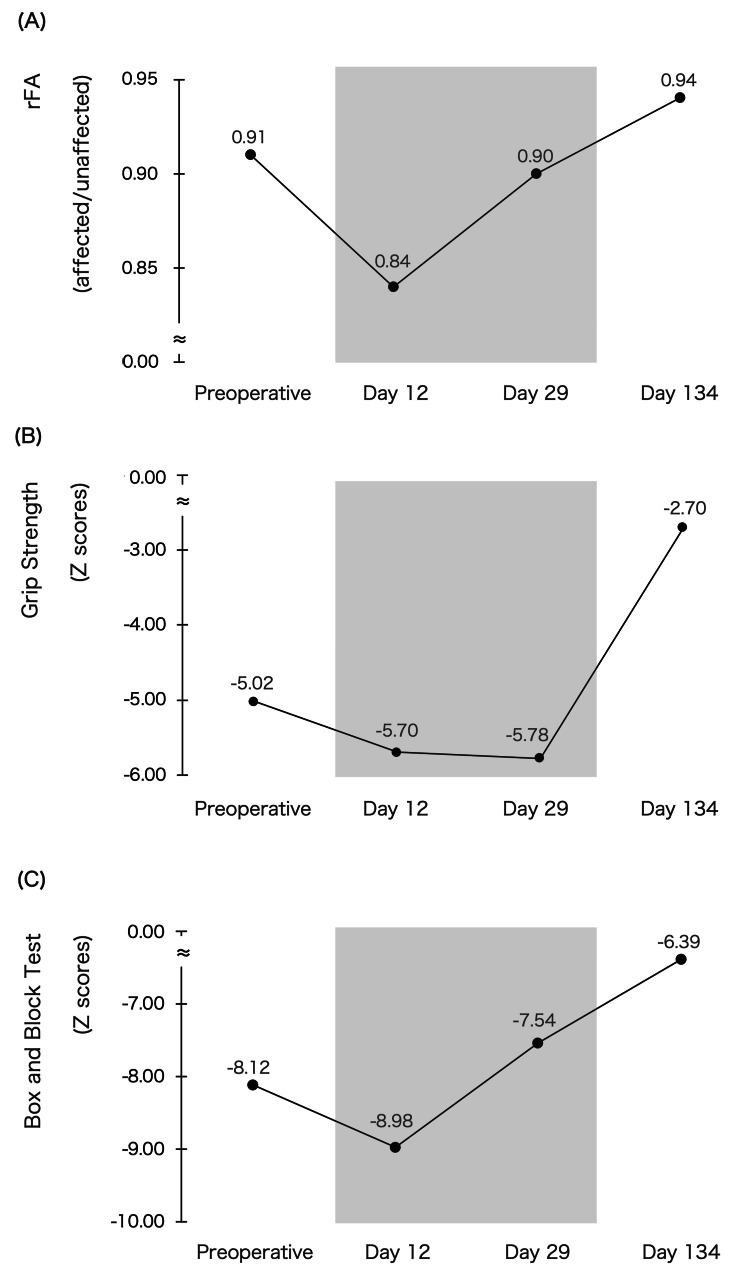
Changes in rFA and motor function (A) relative fractional anisotropy (rFA), (B) Grip Strength Z score, and (C) Box and Block Test Z score. The gray area represents the hospitalization period.

Figure 1 illustrates MRI T2 FLAIR high-intensity areas, tumor regions in T1-weighted images, and CST FA values. Figures 2A-2C display changes in rFA and motor function.

On postoperative day 12, the high-intensity area on MRI T2 FLAIR extended significantly into the frontoparietal lobe. However, by days 29 and 134, the high-intensity region in the frontal lobe had notably decreased. Concurrently, rFA, GS, and BBT Z-scores declined on day 12 but subsequently improved on days 29 and 134. Additionally, on day 134, the patient was able to hang laundry with his paralyzed hand.

## Discussion

This case demonstrates that motor function and rFA transiently declined on postoperative day 12, coinciding with increased edema in MRI T2 FLAIR. Motor function and rFA improved on days 29 and 134, correlating with a reduction in edema. To our knowledge, this is the first study to investigate temporal changes in motor function, rFA, and edema in glioma patients undergoing continuous rehabilitation post-craniotomy.

Evaluating CST integrity using rFA and assessing edema via MRI T2 FLAIR are critical for understanding motor function decline. Previous studies in stroke patients reported that CST edema and rFA influence motor function recovery [8,9]. In glioma patients, an acute phase rFA above 0.80 has been identified as a predictor of postoperative motor recovery [15].

A decrease in rFA may result from vasogenic or peritumoral edema, both of which can be visualized as high-intensity areas on MRI T2 FLAIR [6]. Vasogenic edema-induced CST alterations may be reversible [16]. In our case, the rFA remained above 0.80 postoperatively and increased over time. The concurrent reduction in MRI T2 FLAIR high-intensity areas and motor function improvement suggests that postoperative functional decline was primarily due to vasogenic edema. This finding implies that an rFA above 0.80 may indicate vasogenic edema in glioma patients experiencing motor function decline post-craniotomy.

Continuous rehabilitation is effective in improving motor function post-craniotomy [1-3]. In our case, continuous rehabilitation during hospitalization and post-discharge facilitated motor recovery, consistent with previous findings. Notably, rehabilitation may mitigate motor decline during vasogenic edema resolution. Thus, continuous rehabilitation is essential for glioma patients experiencing postoperative motor deterioration.

Recent studies suggest that continuous rehabilitation may contribute to structural plasticity of the CST as indicated by increased FA [10,11]. Zhang et al. demonstrated that continuous rehabilitation could enhance FA in the CST, indicating improved white matter integrity and motor pathway efficiency [10]. These findings suggest that FA improvements may reflect effective motor re-learning and neuroplastic changes, reinforcing the potential of rehabilitation to facilitate both functional and structural recovery.

In this study, a continuous rehabilitation approach combining mCIMT with occupational therapy, such as stretching and neuromuscular training for the upper limb, likely enhanced the structural plasticity of the CST and promoted motor reorganization. Additionally, the patient's active engagement in daily activities and adherence to the rehabilitation program likely contributed to the successful integration of the affected hand into functional tasks. These results underscore the need for continuous rehabilitation to maximize motor recovery and white matter integrity in glioma patients experiencing postoperative motor deterioration.

This study has limitations. As a single case study, its findings may not be generalizable and limit the ability to perform robust statistical analyses. Additionally, postoperative functional recovery in cases with FA values ≤0.8 remains unverified. Furthermore, while this study primarily focused on FA as a measure of structural plasticity, additional functional measures such as motor evoked potentials and kinematic assessments could provide a more comprehensive understanding of motor recovery. The absence of these measures may limit the relationship between FA and motor function. Despite these limitations, this study provides valuable insights into the dynamic interplay between edema, FA values, and motor function post-craniotomy.

## Conclusions

Continuous rehabilitation plays a crucial role in promoting motor recovery in glioma patients experiencing postoperative decline. This case study highlights the importance of serial assessments of CST integrity using rFA and edema evaluation via MRI T2 FLAIR. The findings suggest that postoperative motor deterioration may be primarily influenced by vasogenic edema, which can be reversible with appropriate rehabilitation strategies.

In clinical practice, integrating CST and edema assessments into postoperative monitoring may help clinicians distinguish between transient and permanent motor deficits, allowing for more targeted rehabilitation interventions. While further studies are needed to validate these findings in larger cohorts, this case underscores the potential of combining imaging biomarkers with structured rehabilitation to optimize functional outcomes in glioma patients undergoing craniotomy.

## References

[1] Burgess G, Jensen LE (2019). Occupational therapy for adults with brain tumors in the acute care setting. NeuroRehabilitation.

[2] Bartolo M, Zucchella C, Pace A (2012). Early rehabilitation after surgery improves functional outcome in inpatients with brain tumours. J Neurooncol.

[3] Yu J, Jung Y, Park J, Kim JM, Suh M, Cho KG, Kim M (2019). Intensive rehabilitation therapy following brain tumor surgery: a pilot study of effectiveness and long-term satisfaction. Ann Rehabil Med.

[4] Fang S, Li Y, Wang Y, Zhang Z, Jiang T (2020). Awake craniotomy for gliomas involving motor-related areas: classification and function recovery. J Neurooncol.

[5] Lu S, Ahn D, Johnson G, Cha S (2003). Peritumoral diffusion tensor imaging of high-grade gliomas and metastatic brain tumors. AJNR Am J Neuroradiol.

[6] Villanueva-Meyer JE, Mabray MC, Cha S (2017). Current clinical brain tumor imaging. Neurosurgery.

[7] Nucifora PG, Verma R, Lee SK, Melhem ER (2007). Diffusion-tensor MR imaging and tractography: exploring brain microstructure and connectivity. Radiology.

[8] Kwak SY, Son SM, Choi BY (2011). Prognostic factors for motor outcome in patients with compressed corticospinal tract by intracerebral hematoma. NeuroRehabilitation.

[9] Puig J, Blasco G, Daunis-I-Estadella J (2013). Decreased corticospinal tract fractional anisotropy predicts long-term motor outcome after stroke. Stroke.

[10] Zhang H, Zhao J, Fan L (2024). Exploring the structural plasticity mechanism of corticospinal tract during stroke rehabilitation based automated fiber quantification tractography. Neurorehabil Neural Repair.

[11] Wen H, Alshikho MJ, Wang Y, Luo X, Zafonte R, Herbert MR, Wang QM (2016). Correlation of fractional anisotropy with motor recovery in patients with stroke after postacute rehabilitation. Arch Phys Med Rehabil.

[12] Mathiowetz V, Volland G, Kashman N, Weber K (1985). Adult norms for the box and block test of manual dexterity. Am J Occup Ther.

[13] Li KY, Lin LJ, Chan AT, Chen CH, Chang WM, Cho YJ (2020). Population based norms for the box and blocks test in healthy right-handed Taiwanese adults. Biomed J.

[14] Morris DM, Taub E, Mark VW (2006). Constraint-induced movement therapy: characterizing the intervention protocol. Eura Medicophys.

[15] Cepeda S, García-García S, Arrese I, Velasco-Casares M, Sarabia R (2021). Acute changes in diffusion tensor-derived metrics and its correlation with the motor outcome in gliomas adjacent to the corticospinal tract. Surg Neurol Int.

[16] Halstead MR, Geocadin RG (2019). The medical management of cerebral edema: past, present, and future therapies. Neurotherapeutics.

